# A One-Step Ultrasonic Spray Pyrolysis Approach to Morphology-Controlled Synthesis of Hollow and Porous YBO_3_:Eu^3+^ Microspheres

**DOI:** 10.3390/nano16130811

**Published:** 2026-06-30

**Authors:** Linhui Gao, Yifu Liu, Hongliang Zhu, Yuan Wang, Qiuying Wang, Xinggang Shan

**Affiliations:** 1 School of Mechanical Engineering, Zhejiang University of Water Resources and Electric Power, Hangzhou 310018, China; gaolhui@zuwe.edu.cn; 2 School of Civil Engineering and Architecture, Keyi College of Zhejiang Sci-Tech University, Shaoxing 312369, China; lyf1571955524@ky.zstu.edu.cn (Y.L.);; 3 School of Materials Science and Engineering, Zhejiang Sci-Tech University, Hangzhou 310018, China

**Keywords:** rare earth phosphors, YBO_3_:Eu^3+^, hollow microspheres, ultrasonic spray pyrolysis

## Abstract

YBO_3_:Eu^3+^ phosphors are regarded as strong candidates for high-performance luminescent materials owing to their excellent luminescence efficiency. In this study, novel YBO_3_:Eu^3+^ porous/hollow microspheres were synthesized via a simple, continuous ultrasonic spray pyrolysis (USP) process using different organic additives. XRD analysis confirms that all samples crystallize in a pure hexagonal YBO_3_ phase, indicating that the additives do not affect phase formation. Electron microscopy reveals a clear morphological evolution from solid to porous and hollow microspheres, with tunable shell thickness and cavity size. Compared with solid microspheres, the obtained hollow microspheres significantly reduce the consumption of rare earth materials with minimal influence on luminescence properties. The results suggest that hollow microspheres are promising substitutes for solid microspheres in the field of rare earth phosphors and the ultrasonic spray pyrolysis (USP) approach shows great potential in large-scale synthesis of morphology-controllable microspheres.

## 1. Introduction

The rare earth borate phosphor YBO_3_:Eu^3+^ exhibits remarkable luminescence intensity under ultraviolet excitation due to its high damage threshold, excellent absorption in vacuum ultraviolet range, and broad emission derived from stable f-f energy level transitions [1]. At present, YBO_3_:Eu^3+^ is widely applied in optical devices such as field emission displays, plasma display panels, and next-generation mercury-free fluorescent lamps [2]. It also demonstrates potential in lighting [3], solar cells [4], biomarkers [5], and medical imaging technologies [6]. With the rapid expansion of these applications, rare earth resources are becoming increasingly scarce worldwide, and greater attention has been directed toward addressing this issue [6,7].

The luminescence properties of phosphors are strongly influenced by particle size and morphology [8]. To date, YBO_3_:Eu^3+^ phosphors with various morphologies have been synthesized, including nanowires/tubes [9], spherical structures [10], and three-dimensional flower-like architectures [11]. Among these, micron- and submicron-scale spherical YBO_3_:Eu^3+^ phosphors (YBO_3_:Eu^3+^ luminescent microspheres) have attracted significant attention due to their high bulk density, favorable slurry characteristics, and low light scattering [12]. Current synthesis methods for YBO_3_:Eu^3+^ luminescent microspheres include hydrothermal synthesis [13], spray drying [14], and co-precipitation [15]. The hydrothermal method produces spherical powders with a narrow particle-size distribution but suffers from a long processing cycle, limiting large-scale production. Spray drying often results in low chromatic purity, while co-precipitation frequently produces compositional inconsistencies, irregular morphologies, and poor dispersion.

The preceding studies describe the recent progress on YBO_3_:Eu^3+^ phosphors, as well as the advantages and limitations of current preparation methods and the challenge of rare earth scarcity [1,15]. Importantly, photoluminescence properties are highly dependent on the surface layer of phosphors [16]. In this context, a novel YBO_3_:Eu^3+^ hollow microsphere was proposed to minimize rare earth usage. To the best of our knowledge, reports on hollow YBO_3_:Eu^3+^ luminescent microspheres remain scarce. Owing to their structural advantages, fluorescent hollow microspheres also show potential for biomedical applications such as drug delivery, biomaterials, water treatment, supercapacitor, etc. [7,12]. Furthermore, rare-earth-doped hollow luminescent microspheres help reduce the consumption of costly rare-earth metals. Their low density enhances dispersion, thereby improving the uniformity and bulk density of fluorescent coating materials [17]. Consequently, the development of simple, cost-effective, and environmentally friendly synthesis strategies for hollow YBO_3_:Eu^3+^ luminescent microspheres has become an important research direction.

Compared with conventional synthesis routes, the USP method offers several distinct advantages for fabricating morphology-controllable microspheres. Hydrothermal synthesis produces narrow particle-size distributions but requires long processing times and high-pressure vessels, limiting large-scale production. Spray drying tends to yield hollow or porous particles with low chromatic purity due to incomplete decomposition. Co-precipitation frequently produces compositional inconsistencies, irregular morphologies, and poor dispersion. In contrast, USP is a one-step, continuous process in which aerosol droplets serve as micro-reactors, enabling simultaneous control over composition, morphology, and crystallinity within seconds [17,18,19]. The method is readily scalable and does not require post-synthesis calcination steps, making it particularly suitable for the synthesis of rare-earth phosphor microspheres with tunable structures.

In this study, a facile and continuous ultrasonic spray pyrolysis (USP) method was employed to synthesize solid, porous, and hollow YBO_3_:Eu^3+^ microspheres by introducing citric acid and sucrose. The formation mechanisms and luminescent properties of the three types of YBO_3_:Eu^3+^ microspheres were systematically investigated. This method also provides valuable insights for synthesizing other porous or hollow microspheres.

## 2. Experimental Part

### 2.1. Experimental Materials and Groups

For consistency, the precursor concentration in ultrasonic spray precursor solutions was fixed at 10 wt.%, and 10 distinct precursor solutions were prepared. The experimental materials used in this work were divided into two categories: inorganic nitrate salts and organic additives (Table 1). Equipment specifications are listed in Table 2. Characterization studies were performed on solid, porous, and hollow YBO_3_:Eu^3+^ microspheres synthesized by the USP method, with the relevant instruments summarized also in Table 2. All chemicals used were of analytical reagent (AR) grade and used without further purification.

This study was divided into three groups: the S group with solid microspheres as the product, the P group with porous microspheres as the product, and the H group with hollow microspheres as the product. The detailed classification is provided in Table 3. Furthermore, the S group prepared four distinct Eu^3+^ molar concentrations—1 mol%, 3 mol%, 5 mol%, and 7 mol%—to systematically investigate the impact of Eu^3+^ ratio; the corresponding samples were designated S1, S3, S5, and S7, respectively. With Eu^3+^ molar concentration fixed at 5 mol%, porous microsphere precursor solutions—designated P1, P3, and P5—were prepared by adding citric acid at concentrations of 5 g/L, 15 g/L, and 25 g/L, respectively, under the same conditions as the S group. Hollow microsphere precursors were prepared under the same conditions as the porous group, except sucrose was used as the additive at concentrations of 5 g/L, 15 g/L, and 25 g/L. The resulting products were designated H1, H3, and H5, respectively.

### 2.2. Preparation of YBO_3_:Eu^3+^ Microspheres

YBO_3_:Eu^3+^ microspheres were successfully synthesized via the ultrasonic spray pyrolysis (USP) method. Taking sample S1 as an example, 17.07 g of Y(NO_3_)_3_·6H_2_O, 2.81 g of H_3_BO_3_, and 0.18 g of Eu(NO_3_)_3_·6H_2_O were dissolved in 180 mL of deionized water. The solution was magnetically stirred for 30 min to form a precursor. The precursor solution was then atomized in an ultrasonic generator (1.7 MHz), producing droplets that were carried by a gas stream into an 800 °C tube furnace. In the furnace, solvent evaporated rapidly from the droplet surfaces. As the reaction progressed, the precursor decomposed into metal oxides and nitrogen oxide gases, which were discharged through a scrubber bottle. Finally, YBO_3_:Eu^3+^ microspheres were collected in a quartz vessel.

**Scheme 1:**
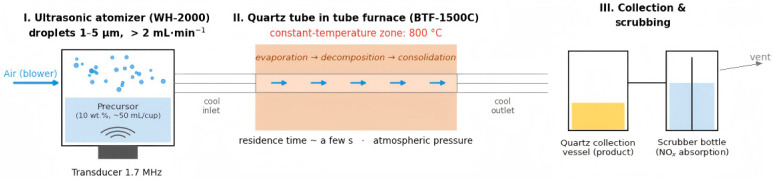
Schematic illustration of the ultrasonic spray pyrolysis apparatus.

A schematic diagram of the USP apparatus is presented in Scheme 1. The set-up consists of three sections connected in series. (i) The atomization unit is a 1.7 MHz ultrasonic nebulizer (WH-2000) equipped with a 50 mL solution cup and a built-in adjustable blower; at this excitation frequency the device generates aqueous mist droplets in the 1–5 μm size range (number-mean ≈ 3 μm, consistent with the Lang equation [19]), and the precursor solution is fed into the aerosol stream at a nebulization rate of >2 mL·min^−1^. (ii) The aerosol is transported by the blower-supplied air, which acts as both the carrier and the oxidizing gas, through a horizontal quartz tube mounted in the tube furnace (BTF-1500C). The quartz tube passes through the furnace so that only its central portion lies within the constant-temperature zone held at 800 °C, while the inlet and outlet ends remain near ambient temperature; the droplets therefore experience rapid heating on entering the hot zone and are quenched on leaving it. Quartz (softening point > 1270 °C) is chemically inert and fully stable at the 800 °C processing temperature. (iii) The collection section comprises a quartz collection vessel in which the solid product is gathered, followed by a water-filled scrubber bottle that absorbs the nitrogen oxide off-gases released by nitrate decomposition before the gas stream is vented. The whole system operates as a one-pass, continuous, open flow at atmospheric pressure; with a carrier-gas flow of the order of a few liters per minute through a tube of this size, the droplet/particle residence time within the heated zone is of the order of a few seconds, which is characteristic of laboratory-scale USP reactors and is sufficient for complete solvent evaporation, precursor decomposition, and particle consolidation [19,20].

The surface morphology of the samples was observed using field emission scanning electron microscopy (FESEM). Elemental composition and distribution were analyzed by mapping spectroscopy. The crystal structures of the samples were characterized using X-ray diffraction (XRD) with Cu-Kα radiation in the scanning range of 10–80°. Nitrogen adsorption–desorption isotherms were measured using a physical adsorption analyzer to determine the specific surface area and pore size distribution of the samples. The measurements were performed after degassing at 200 °C for 200 min. Photoluminescence (PL) properties were evaluated using a fluorescence spectrophotometer at room temperature. For the gas adsorption measurement, the powder was loaded into the sample tube and degassed as described above. For the PL measurements, equal masses of the different powders were weighed on the same analytical balance and loosely packed, without pelleting, into the same solid-sample holder; each powder was gently leveled with a glass slide following an identical procedure, so that the filled volume of the holder cavity—and hence the apparent volumetric packing density—was kept comparable across all samples. All PL spectra were recorded in a single session under identical instrument settings (excitation/emission slit widths, detector voltage, and scan parameters). The Eu^3+^ contents quoted in this work are nominal values based on the precursor stoichiometry. Because in spray pyrolysis each droplet acts as a closed micro-reactor that retains the complete cation inventory of the precursor solution—there is no precipitation, filtration, or washing step in which selective cation loss could occur—the composition of USP-derived particles is generally accepted to follow the precursor stoichiometry closely [17,19].

## 3. Results and Discussion

### 3.1. Material Phase Analysis

**Figure 1:**
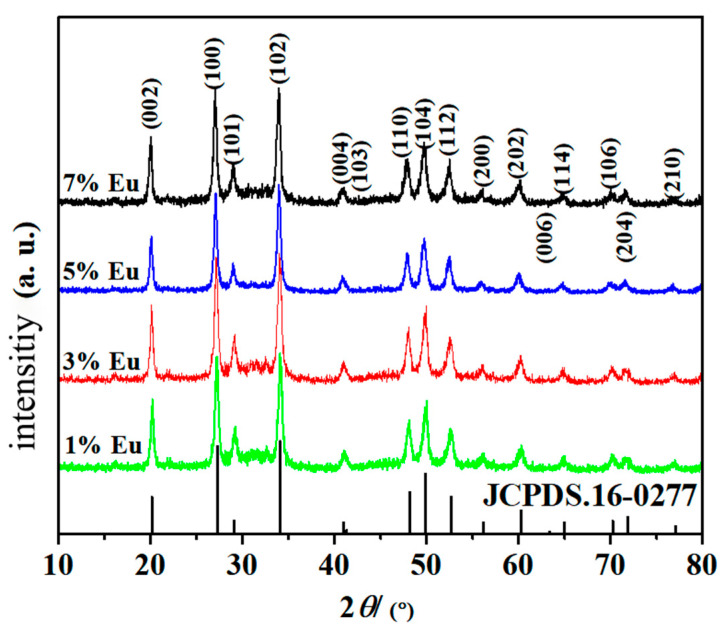
XRD patterns of YBO_3_:Eu^3+^ samples with different Eu^3+^ concentrations.

Figure 1 shows the XRD patterns of solid YBO_3_:Eu^3+^ microspheres with different Eu^3+^ concentrations (1 mol%, 3 mol%, 5 mol%, and 7 mol%) synthesized via the USP method at 800 °C. All four samples exhibit sharp diffraction peaks, indicating excellent crystallinity of YBO_3_:Eu^3+^ phosphors. The peaks fully coincide with those of the standard YBO_3_ reference card (JCPDS 16–0277), with no extraneous peaks detected. This confirms that the USP method produces pure hexagonal YBO_3_:Eu^3+^ without impurities.

**Figure 2:**
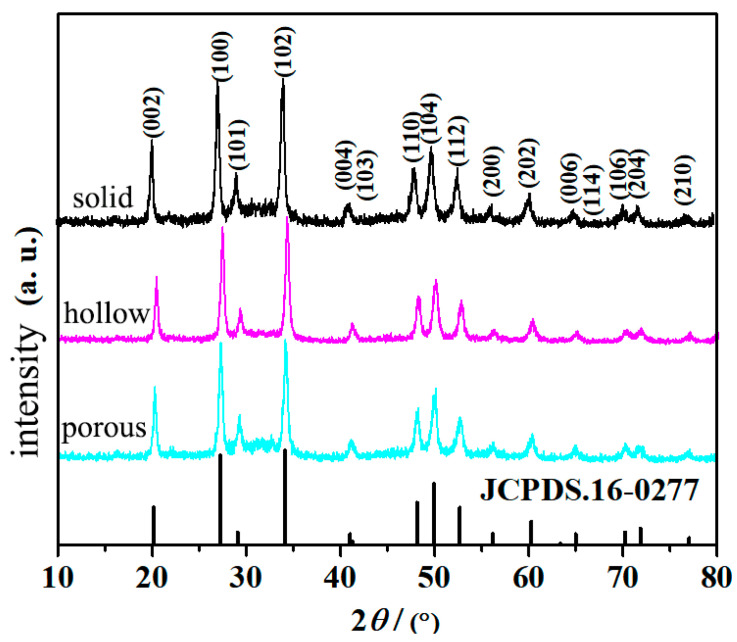
XRD patterns of YBO_3_:Eu^3+^ samples with three morphologies.

To further examine the effects of organic additives on the phase composition, XRD patterns of solid, porous, and hollow YBO_3_:Eu^3+^ samples (S5, P3, and H3 in Table 3) prepared by the USP method at 800 °C are shown in Figure 2. All three samples exhibit sharp diffraction peaks, again confirming excellent crystallinity. The diffraction patterns of the different morphologies are essentially identical and fully consistent with the YBO_3_ standard card (JCPDS 16-0277), with no background peaks. These results indicate that the addition of organic additives did not significantly affect the crystallinity of the samples.

### 3.2. Morphology

**Figure 3:**
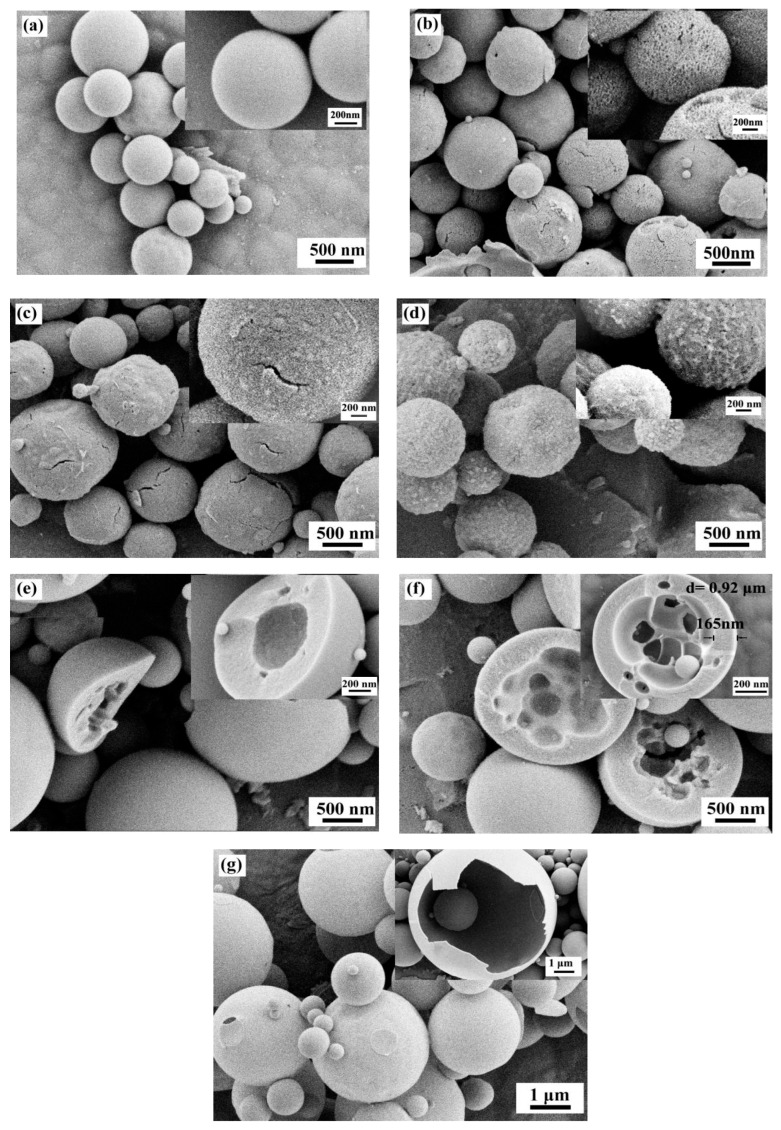
Ultrasonic spray products YBO_3_:Eu^3+^ FE-SEM: (**a**) solid microspheres S5; (**b**) porous microspheres P1; (**c**) porous microspheres P3; (**d**) porous microspheres P5; (**e**) hollow microspheres H1; (**f**) hollow microspheres H3; (**g**) hollow microspheres H5.

To investigate the effects of organic additives on morphology, solid, porous, and hollow YBO_3_:Eu^3+^ microspheres synthesized by the ultrasonic spray method were analyzed. Figure 3a–g present FESEM images of YBO_3_:Eu^3+^ samples. All samples exhibit regular spherical morphology with particle sizes in the micrometer range and demonstrate good dispersibility without significant agglomeration. Figure 3a depicts the FESEM image of solid sample S5, displaying an intact spherical structure with a smooth surface and uniform dimensions.

Figure 3b–d show porous YBO_3_:Eu^3+^ microspheres P1, P3, and P5. These particles have comparable sizes and porous structures. As shown in the insets, the external pores enlarge with increasing citric acid content. This occurs because, during ultrasonic spray pyrolysis, droplets generated by the atomizer undergo rapid dehydration and pyrolysis in the high-temperature tube furnace. The carboxyl groups of citric acid chelate with metal ions in the precursor solution, forming stable complexes that are uniformly dispersed within the droplets. As the reaction proceeds and metal ions form YBO_3_:Eu^3+^, citric acid decomposes at high temperatures. Consequently, higher citric acid concentrations lead to larger pores [21].

**Figure 4:**
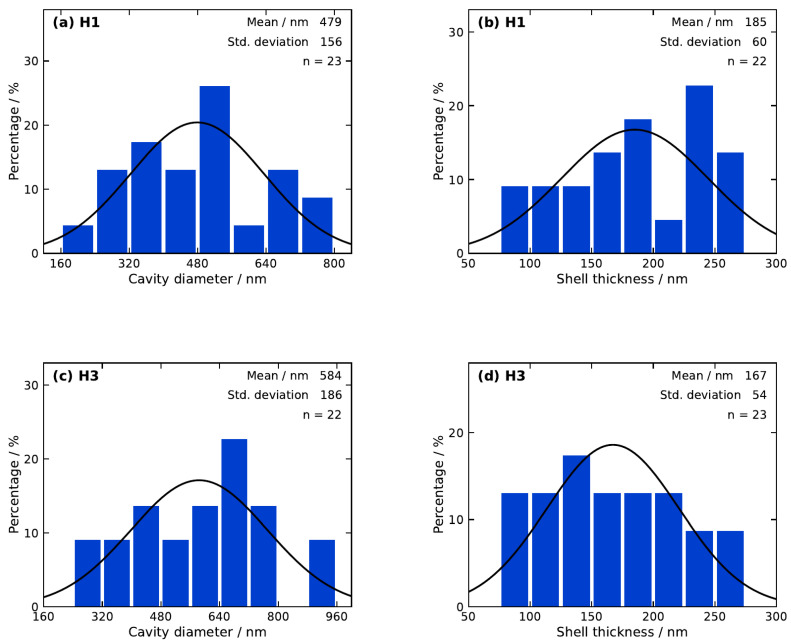
Size distributions of cavity diameter (**a**,**c**) and shell thickness (**b**,**d**) of hollow YBO_3_:Eu^3+^ microspheres for samples H1 (**a**,**b**) and H3 (**c**,**d**), measured from FESEM cross-sectional images (*n* = 23 and 22, respectively). Solid curves represent Gaussian fits to the distributions.

Figure 3e–g reveal hollow YBO_3_:Eu^3+^ microspheres. To quantify the size uniformity of the hollow structures, ImageJ (National Institutes of Health, Bethesda, MD, USA) was used to analyze FESEM cross-sectional images of samples H1 and H3. To expose the internal structure, a portion of each sample was lightly ground using an agate mortar prior to FESEM observation. For particles with clearly visible cross-sections, the cavity-to-outer-diameter ratio was directly measured (H1: 0.588 ± 0.012, *n* = 5; H3: 0.615 ± 0.034, *n* = 5), and this ratio was then applied to all observable microspheres to estimate cavity diameters. Statistical analysis of H1 (*n* = 23) yielded a mean cavity diameter of 479 ± 156 nm and a mean shell thickness of 185 ± 60 nm; for H3 (*n* = 22), the corresponding values were 584 ± 186 nm and 167 ± 54 nm, respectively. The size distributions of cavity diameter and shell thickness for H1 and H3 are presented in Figure 4a–d, with Gaussian fits overlaid on each histogram. Both distributions follow approximately Gaussian profiles, confirming the structural uniformity of the hollow microspheres. The larger cavity diameter and thinner shell of H3 compared to H1 are consistent with the higher sucrose concentration in the H3 precursor solution. This trend extends across the full H-series: the images also clearly show a gradual increase in hollow volume from samples H1 to H5. Cross-sectional images demonstrate progressively thinner walls, attributed to the increasing sucrose content in the precursor solution. During ultrasonic spray pyrolysis, droplets formed by high-frequency resonance undergo rapid dehydration and pyrolysis. As heating continues, a thin metal-ion film forms on the droplet surfaces while insoluble nanoparticles migrate toward the droplet surface, leaving internal [22].

**Figure 5:**
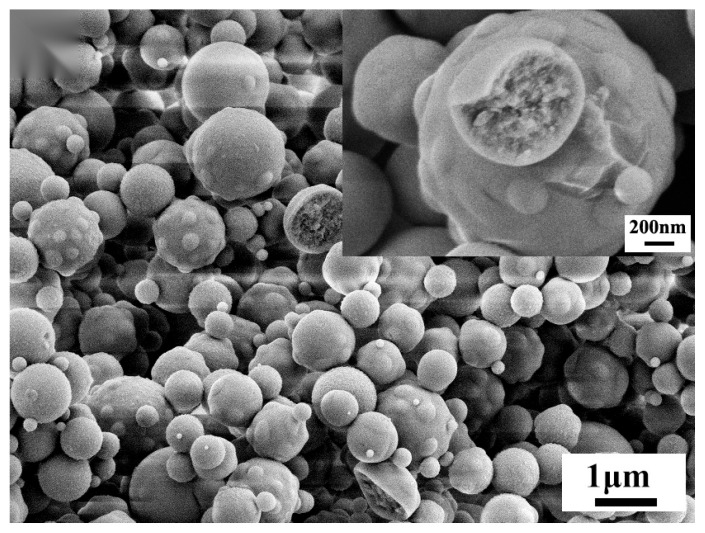
FESEM image of the ground porous YBO_3_:Eu^3+^ sample P3; the inset shows a fractured microsphere whose interior is completely filled with primary nanocrystallites, with no central cavity.

To verify that the porous microspheres are non-hollow in nature, a portion of the P3 sample was ground in the same manner and examined by FESEM (Figure 5). The fractured spheres exhibit interiors that are completely filled with aggregated primary nanocrystallites, and no central cavity is observed in any of the fractured particles. The granular fracture texture further indicates that the voids between the primary crystallites in the interior are largely closed and poorly interconnected, which is consistent with the very low N_2_ uptake discussed in Section 3.3. The S-samples, prepared without any gas-evolving additive, display smooth and dense surfaces (Figure 3a), and no broken-shell fragments—which are abundant and characteristic in the ground H-samples—were observed in the S-sample images, supporting their solid nature; mechanistically, cavity formation is not expected in the absence of an additive (Section 4). It should nevertheless be noted that SEM probes the local texture of a finite number of particles. In this work, the cross-sectional statistics of the H-samples were collected from particles fractured at random positions by grinding (*n* = 23 and 22 for H1 and H3), which samples the powder considerably more representatively than isolated single-particle images; even so, bulk techniques such as TEM or mercury intrusion porosimetry would provide further independent confirmation, and such measurements are planned as future work.

### 3.3. Specific Surface Area and Pore Size Distribution Analysis

**Figure 6:**
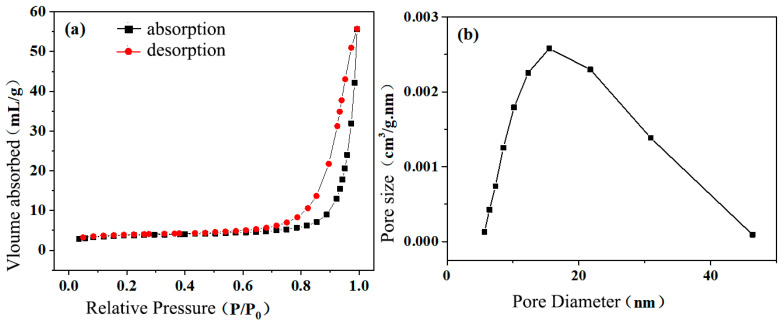
Porous YBO_3_:Eu^3+^ microspheres: (**a**) N_2_ adsorption–desorption isotherm; (**b**) pore size distribution.

Figure 6a shows the N_2_ adsorption–desorption isotherm of the P3 sample. The isotherm is of Type III with a very low overall N_2_ uptake, which is characteristic of an essentially non-porous (or macroporous) solid with weak adsorbent–adsorbate interactions [23]. The steep increase in adsorption at relative pressures approaching P/P_0_ = 1, accompanied by a narrow H3-type hysteresis loop, most likely arises from gas condensation on the external particle surfaces and in the interstitial cavities between the small packed particles, a behavior typical of finely powdered samples, rather than from capillary condensation within intraparticle mesopores. The specific surface area of P3, determined by the BET method, is 16.08 m^2^/g, higher than that expected for smooth dense spheres of comparable diameter, which is consistent with the rough, crater-like surface texture observed by FESEM (Figure 3b–d). The pore size distribution shown in Figure 6b, calculated from the isotherm using the BJH model, exhibits an apparent maximum at about 28 nm in the 8–47 nm range; however, because the BJH analysis presupposes the existence of intraparticle mesopores, a distribution obtained from such raw data should be interpreted with caution and may largely reflect interparticle voids rather than true internal pores. Accordingly, the gas-adsorption data provide no firm evidence that the spherical particles themselves are internally mesoporous. Throughout this work, the term “porous” is therefore used in a morphological sense, referring to the open, crater-like surface texture of the P-samples revealed by FESEM, rather than implying a developed intraparticle pore network in the bulk.

Nevertheless, the surface texture of P3 is expected to influence its luminescence. The larger external surface area introduces more surface defects and dangling bonds, which act as non-radiative recombination centers and quench the luminescence of rare-earth phosphors [9,24]. In addition, the rough, crater-like surface enhances the scattering of both the excitation and emission light, and residual carbonaceous species originating from the incomplete combustion of citric acid may absorb part of the ultraviolet excitation. These surface-related factors together provide a more plausible explanation for the lower luminescence intensity of the P3 sample (Section 3.4) than intraparticle mesoporosity. For a quantitative comparison in the absence of measured isotherms for the solid and hollow samples, their surface areas can be estimated geometrically: for smooth dense spheres of ~1 μm diameter and a density of ≈4.4 g·cm^−3^ (hexagonal YBO_3_), the geometric specific surface area is only ~1.4 m^2^/g, and for the H3 hollow spheres (outer diameter ≈ 918 nm, shell thickness ≈ 167 nm; Section 3.2) it amounts to ~2–3 m^2^/g even when the inner shell surface is included. The measured surface area of P3 (16.08 m^2^/g) is therefore roughly an order of magnitude larger than those expected for the S- and H-samples, which quantitatively supports the surface-area-based interpretation of the PL differences discussed in Section 3.4.

### 3.4. Luminescence Performance Analysis

**Figure 7:**
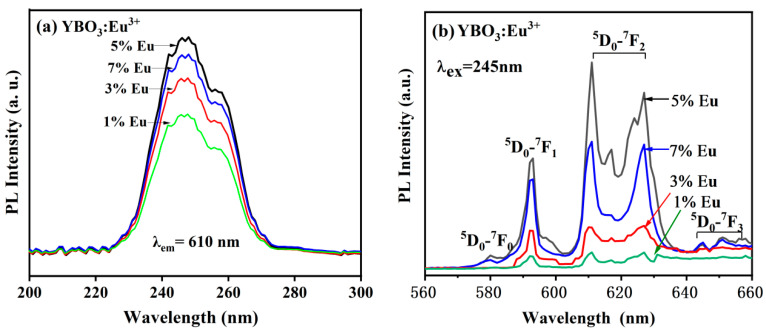
Fluorescence spectra of YBO_3_:Eu^3+^ phosphors with different Eu^3+^ contents: (**a**) Excitation spectra; (**b**) Emission spectra.

PL spectra of YBO_3_:Eu^3+^ microspheres synthesized by the USP method with varying Eu^3+^ concentrations are shown in Figure 7. Figure 7a presents the excitation spectrum monitored at 610 nm, showing a broad excitation band between 220–280 nm with maximum intensity at 245 nm. The highest luminescence intensity was obtained at 5 mol% Eu^3+^ doping. The emission intensity of S7 is notably lower than that of S5. All spectra in Figure 7 were recorded in a single session with the identical sample-loading protocol described in Section 2.2, so that instrumental drift and preparation-related variations were minimized; moreover, the intensities vary systematically and non-monotonically across the concentration series (S1 < S3 < S5 > S7), a pattern that random experimental error would not produce. The decrease beyond 5 mol% is fully consistent with the concentration quenching widely reported for YBO_3_:Eu^3+^, whose optimum doping level lies near 5 mol% [4,15]: at higher Eu^3+^ contents, the average Eu–Eu distance is reduced, promoting non-radiative cross-relaxation energy transfer between adjacent Eu^3+^ ions. Figure 7b shows the emission spectrum under 245 nm excitation, revealing three main emission bands between 570–660 nm. Peaks at 580, 592, and 650 nm correspond to the ^5^D_0_→^7^F_0_, ^5^D_0_→^7^F_1_, and ^5^D_0_→^7^F_3_ [25]. The strongest peak at 612–618 nm corresponds to the ^5^D_0_→^7^F_2_ transition, which gives rise to a pure red emission with high chromatic purity. Compared with conventional methods that yield orange emission dominated by the ^5^D_0_→^7^F_1_ transition, USP-prepared YBO_3_:Eu^3+^ demonstrates superior red emission [26]. These findings highlight the industrial potential of the USP method for producing high-performance microsphere phosphors.

All PL measurements were performed using the same sample mass (equal-mass basis) to ensure consistency across morphologies. It should be noted that, owing to their hollow interior, hollow microspheres contain less rare-earth material per particle than solid microspheres of comparable outer diameter. Consequently, an equal-mass comparison effectively employs a greater number of hollow particles per measurement.

**Figure 8:**
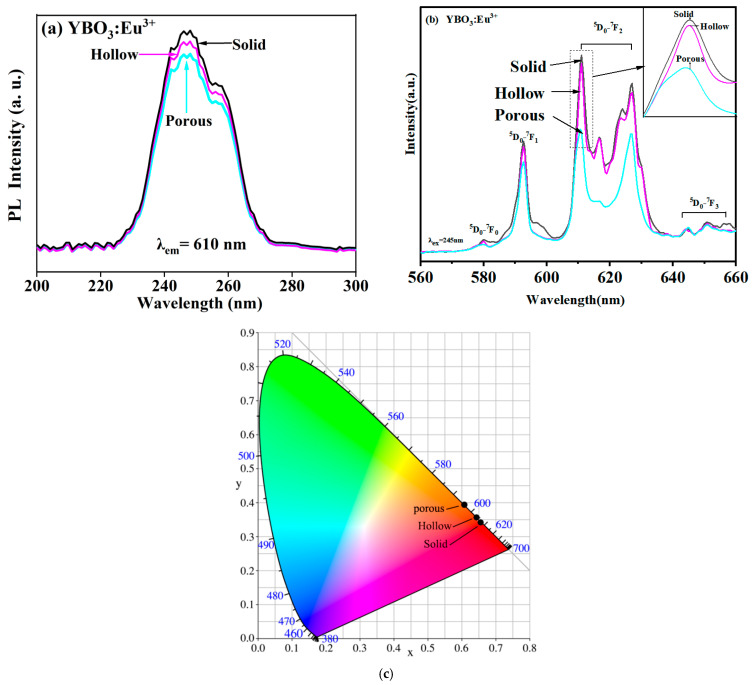
Fluorescence spectra of YBO_3_:Eu^3+^ phosphors with different morphologies: (**a**) Excitation spectra; (**b**) Emission spectra; (**c**) CIE chromaticity diagram.

To investigate the influence of morphology on luminescence at the optimal doping level, the Eu^3+^ content of the porous and hollow samples was fixed at the optimal value of 5 mol% determined above. Note that the numerical suffixes of the P- and H-series denote the additive concentration (5, 15, and 25 g/L; Section 2.1), not the Eu^3+^ content; S5, P3, and H3 thus all contain the same optimal Eu^3+^ content (5 mol%), and any difference in their PL therefore reflects the change in morphology rather than a change in Eu^3+^ concentration. Under identical experimental conditions, the excitation and emission spectra of solid, porous, and hollow YBO_3_:Eu^3+^ samples (S5, P3, and H3) were compared, as shown in Figure 8. Figure 8a reveals that the excitation intensities of solid and hollow samples differ only slightly, while the porous sample exhibits somewhat lower excitation intensity. The emission spectra in Figure 8b show a similar trend, with the inset confirming nearly equivalent emission intensities for solid and hollow samples, though the porous sample remains weaker. The comparable luminescence intensities of the solid and hollow samples measured on an equal-mass basis indicate that the hollow shell material retains luminescence activity throughout its thickness, and that the hollow interior does not introduce significant additional non-radiative recombination pathways. The near-unity PL intensity ratio of H3 to S5 (95.82%) on an equal-mass basis suggests that the hollow structure does not introduce significant additional non-radiative recombination centers, consistent with the reported fluorescence lifetime of YBO_3_:Eu^3+^ phosphors (~1 ms for the ^5^D_0_ level), which is characteristic of a radiative-dominated decay process [25]. The weaker emission of the porous sample P3 is consistent with its larger external specific surface area (16.08 m^2^/g, Section 3.3) and rough, crater-like surface texture, which introduce a higher density of surface defects acting as non-radiative recombination centers, enhance the scattering of the excitation and emission light, and may involve residual carbonaceous species absorbing part of the ultraviolet excitation, thereby reducing the luminescence efficiency [9,24]. Table 4 presents the detailed strongest emission peaks for each morphology of YBO_3_:Eu^3+^ samples, indicating a slight intensity reduction in the hollow sample compared to the solid sample. Comprehensive FESEM analysis, as illustrated in Figure 3f, reveals that hollow YBO_3_:Eu^3+^ microspheres achieve 95.82% of the luminescence intensity exhibited by solid samples, while concurrently reducing raw material consumption by approximately 26%, estimated from the ratio of hollow cavity volume to total particle volume. Assuming spherical geometry and uniform shell density identical to that of solid microspheres, the material savings ratio *η* is defined as:

*η* = (*d*_cavity_/*d*_outer_)^3^ (1)

where *d*_cavity_ and *d*_outer_ denote the mean cavity diameter and mean outer diameter of H3 microspheres, respectively. Using the statistical mean values from FESEM cross-sectional image analysis (*n* = 22; d_cavity_ = 584 nm, d_outer_ = d_cavity_ + 2 × shell thickness = 584 + 2 × 167 = 918 nm; Section 3.2), Equation (1) gives η = (584/918)^3^ ≈ 26%.

Figure 8c presents the CIE chromaticity diagram of YBO_3_:Eu^3+^ microspheres with different morphologies by CIE 1931 color space. The porous microspheres exhibit chromaticity coordinates in the orange-red region, while both hollow and solid microspheres cluster in the red region. This indicates that solid and hollow microspheres exhibit superior chromatic purity compared to porous counterparts, with solid and hollow samples showing nearly identical chromaticity. These findings confirm that hollow microspheres can effectively replace solid ones, reducing rare earth usage without significant loss in luminescence intensity. The CIE diagram provides complementary information not directly readable from the emission spectra: it visually confirms that the transition from solid to hollow morphology does not compromise chromatic purity, while the porous morphology exhibits a measurable red-to-orange shift that may be associated with its higher surface defect density and altered local Eu^3+^ crystal field environment.

## 4. Mechanism

**Figure 9:**
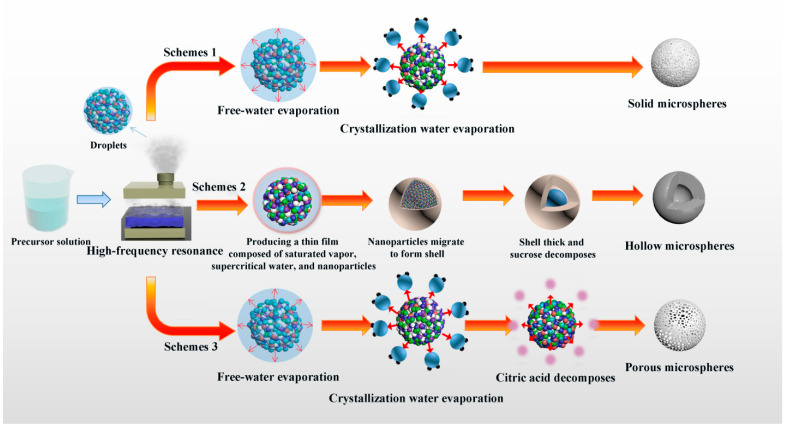
Mechanism diagram of the formation of ultrasonic spray products with different morphologies.

Figure 9 illustrates the formation mechanisms of luminescent microspheres synthesized by ultrasonic spray pyrolysis. Routes ①, ②, and ③ correspond to solid, hollow, and porous microspheres, respectively. When the uniformly mixed precursor solution is atomized into mist droplets (several to tens of micrometers) by high-frequency resonance and enters the high-temperature tube furnace, the reaction pathways differ depending on whether the droplets contain no organic additive, sucrose, or citric acid.

Route ① depicts the formation of solid YBO_3_:Eu^3+^ microspheres. In the high-temperature furnace, droplets self-assemble into spherical structures under surface tension. Through evaporation, decomposition, sintering, and aggregation, they ultimately form solid microspheres.

Route ② shows the formation of hollow YBO_3_:Eu^3+^ microspheres. When sucrose-containing droplets enter the furnace, several coupled physical and chemical mechanisms drive the formation of the hollow structure. First, the high viscosity imparted by dissolved sucrose retards solvent evaporation and suppresses rapid inward diffusion of metal ions, preventing premature precipitation in the droplet interior. Simultaneously, the multiple hydroxyl groups of sucrose form extensive hydrogen bonds with water molecules, further slowing the drying rate and establishing a concentration gradient that drives insoluble YBO_3_ [27]. As a result, a thin, dense shell rich in insoluble nanoparticles forms at the droplet periphery at an early stage [20], while the interior remains largely solute-depleted. As heating continues, sucrose undergoes thermal decomposition above approximately 190 °C, releasing CO_2_ and H_2_ [28]. The carbonaceous residue produced during sucrose pyrolysis transiently stabilizes the inner shell surface, providing mechanical support that prevents shell collapse prior to complete combustion at higher furnace temperatures; this carbon-mediated stabilization role has been documented in analogous sucrose-assisted spray pyrolysis systems for metal oxide hollow structures [18,29]. The final product is a hollow YBO_3_:Eu^3+^ [27].

Route ③ explains the formation of porous YBO_3_:Eu^3+^ microspheres. With citric acid present, its carboxyl groups chelate metal ions, generating organic-inorganic composite droplets. Free water decomposes first at high temperatures. When water content falls below a critical value, droplets enter the falling-rate drying phase. Crystallization water then evaporates while citric acid decomposes into H_2_O and CO_2_. This decomposition process creates pores, resulting in porous YBO_3_:Eu^3+^ microspheres [30].

The feasibility of the proposed multistage mechanism can be assessed by comparing the characteristic time scales involved. In laboratory-scale USP reactors of the type used in this work (Scheme 1), the droplet/particle residence time in the heated zone is of the order of a few seconds [19,20]. By contrast, the evaporation of a water droplet a few micrometers in diameter at such temperatures is completed within milliseconds to a few tens of milliseconds, and the subsequent decomposition of the nitrate precursors and of the organic additives also proceeds on the sub-second scale [19,20]. Likewise, the diffusion time of solute species across a droplet of radius r ≈ 1–2 μm, estimated as t ≈ r^2^/D with a liquid-phase diffusivity D ≈ 10^−9^ m^2^·s^−1^, is of the order of milliseconds. The mass transport required for the solute redistribution and shell formation depicted in Route ② is therefore two to three orders of magnitude faster than the residence time in the heated zone, so the formation of an anisotropic (surface-enriched) structure from an initially homogeneous droplet is kinetically feasible within the time available in the reactor [19,20].

The distinct morphological outcomes induced by citric acid and sucrose can be attributed to their fundamentally different interaction modes with metal ions and their contrasting physicochemical behaviors during spray pyrolysis. Citric acid acts as a chelating agent, forming stable metal–organic complexes with Y^3+^ and Eu^3+^ ions via its carboxyl groups. These complexes are uniformly distributed throughout the droplet interior, so that when citric acid decomposes at high temperature (releasing CO_2_ and H_2_O), pores are generated homogeneously throughout the particle volume, yielding porous microspheres [30]. In contrast, sucrose does not chelate metal ions but instead increases the solution viscosity and preferentially concentrates at the droplet surface during drying, promoting the formation of a dense shell at the droplet periphery while the interior remains depleted [27]. It should be acknowledged that the thermolysis of citric acid and that of sucrose occur in a similar temperature range, both starting at about 180 °C [28], so the decomposition temperature alone cannot account for the different morphologies. Instead, a simpler scheme based on where the organic species and the gas evolution are located within the drying droplet explains the experimental observations. In sucrose-containing droplets, the non-chelating sucrose is enriched together with the solute at the droplet periphery during the rapid surface evaporation, so that gas evolution occurs at, and is confined by, an already-forming surface crust; the trapped gases inflate the particle and a smooth hollow sphere results. In citric-acid-containing droplets, the metal–citrate complexes are distributed throughout the droplet volume, so that gas evolution occurs homogeneously inside the particle while no early rigid crust is available to sustain a single internal cavity; the distributed gas release instead produces the open, crater-like (“porous”) surface texture, and the partial shrinkage of the gas-evolving interior gives the crumpled appearance. In this picture, the decisive factor is the spatial distribution of the organic phase—surface-enriched (sucrose) versus volume-distributed (citric acid)—rather than the onset temperature of its decomposition. We note that the detailed in situ verification of these droplet-scale processes goes beyond the scope of the present study, and the scheme proposed here is the simplest one consistent with all of the experimental observations.

## 5. Conclusions

In this study, solid, hollow, and porous YBO_3_:Eu^3+^ microspheres with pure hexagonal crystal structures and uniform size distributions were successfully synthesized by the ultrasonic spray pyrolysis (USP) method. The mechanisms responsible for the formation of different morphologies were discussed. Among the samples, solid microspheres showed the highest luminescence intensity, followed by hollow microspheres, with porous microspheres showing the weakest. Remarkably, hollow microspheres achieved 95.82% of the luminescence intensity of solid ones while consuming about 26% less raw material, estimated from the volume ratio (d_cavity/d_outer)^3^ based on FESEM measurements (Section 3.2), highlighting their potential as an effective alternative to solid microspheres.

## Figures and Tables

**Table 1:** Raw materials for the experiments.

Name of Raw Material	Chemical Formula	Specifications	Manufacturer
yttrium nitrate	Y(NO_3_)_3_·6H_2_O	AR	Shanghai McLean Biochemical Technology Co., LTD (Shanghai, China)
boric acid	H_3_BO_3_	AR	Xilong Science Co., LTD (Shantou, China)
europium nitrate	Eu(NO_3_)_3_·6H_2_O	AR	Aladdin Reagent (Shanghai) Co., LTD (Shanghai, China)
sucrose	C_12_H_22_O_11_	AR	Xilong Science Co., LTD (Shantou, China)
citric acid in water	C_6_H_8_O_7_	AR	Shanghai McLean Biochemical Technology Co., LTD (Shanghai, China)

**Table 2:** Equipment used in the experiment and characterization of this chapter.

Name	Model	Manufacturer
Vacuum drying oven	DZF-6020	Shanghai Kuntian Instrument Co., LTD (Shanghai, China)
Electronic balance	YP-C2003	Shanghai Guangzheng Medical Instrument Co., LTD (Shanghai, China)
Quartz crucible	-	-
Ultrasonic spray apparatus	WH-2000	Guangdong Yuehua Medical Equipment Factory Co., LTD (Shantou, China)
Electric thermal constant temperature fan drying chamber	DGG-903B	Shanghai Senxin Experimental Instrument Co., LTD (Shanghai, China)
Tube furnace	BTF-1500C	Anhui Beikai Equipment Technology Co., LTD (Hefei, China)
Magnetic heating stirrer	CJJ78-1	Shanghai Meixiang Instrument Co., LTD (Shanghai, China)
X-ray diffractometer	ARL-XTRA	Thermo ARL Company (Ecublens, Switzerland)
Field emission scanning electron microscope	ULTRA-55	Germany Zeiss (Oberkochen, Germany)
Physical adsorption apparatus	ASAP2020HD88	Micrometric Company (Lincolnshire, UK)
Fluorescence spectrometer	F-4600	Hitachi, Ltd. (Macquarie Park, NSW, Australia)

**Table 3:** YBO_3_:Eu^3+^ product classification.

Group	Product	Organic Addition Agent	Effect of Organic Additives
S	Solid	not have	not have
P	Porous	citric acid	pore forming material
H	Hollow	sucrose	film-forming agent

**Table 4:** The strongest emission peak of YBO_3_:Eu^3+^ microspheres at 245 nm excitation.

Sample Name	Solid	Hollow	Porous
The emission peak intensity at 610 nm (a.u.)	1,460,000	1,399,117	922,116

## Data Availability

All data can be found in this manuscript.
